# Bandgap Tuning in Cobalt-Doped BiFeO_3_/Bi_25_FeO_40_ Heterostructured Nanopowders via Sol–Gel Phase Engineering

**DOI:** 10.3390/nano15120918

**Published:** 2025-06-12

**Authors:** Dhouha Baghdedi, Asma Dahri, Mohamed Tabellout, Najmeddine Abdelmoula, Zohra Benzarti

**Affiliations:** 1 Laboratory of Multifunctional Materials and Applications (LaMMA), Faculty of Sciences of Sfax, University of Sfax, BP 1171, Sfax 3000, Tunisia; baghdedidhouha@gmail.com (D.B.); asma1.dhahri1@gmail.com (A.D.); najmeddine.abdelmoula@fss.rnu.tn (N.A.); 2 Institut des Molécules et Matériaux du Mans, UMR CNRS 6283, Le Mans Université, Avenue Olivier Messiaen, 72085 Le Mans Cedex 9, France; mohamed.tabellout@univ-lemans.fr; 3 CEMMPRE, ARISE, Department of Mechanical Engineering, University of Coimbra, Rua Luís Reis Santos, 3030-788 Coimbra, Portugal

**Keywords:** BiFeO_3_, Co doping, sol–gel synthesis, bandgap tuning, multiferroics

## Abstract

Bismuth ferrite (BiFeO_3_, BFO) is a promising multiferroic material, but its optoelectronic potential is limited by a wide bandgap and charge recombination. Here, we report the sol–gel synthesis of Co-doped BiFeO_3_/Bi_25_FeO_40_ heterostructured nanopowders (x = 0.07, 0.15) alongside pristine BFO to explore Co doping and phase engineering as strategies to enhance their functional properties. Using X-ray diffraction (XRD) with Rietveld refinement, Fourier-transform infrared spectroscopy (FTIR), field-emission scanning electron microscopy (FE-SEM), UV-Vis spectroscopy, and dielectric analysis, we reveal a biphasic structure (rhombohedral R3c and cubic I23 phases) with tuned phase ratios (~73:27 for x = 0.07; ~76:24 for x = 0.15). Co doping induces lattice strain and oxygen vacancies, reducing the bandgap from 1.78 eV in BFO to 1.31 eV in BFO_0.15_ and boosting visible light absorption. Dielectric measurements show reduced permittivity and altered conduction, driven by [Co^2+^-V_0_^••^] defect dipoles. These synergistic modifications, including phase segregation, defect chemistry, and nanoscale morphology, significantly enhance optoelectronic performance, making these heterostructures compelling for photocatalytic and photovoltaic applications.

## 1. Introduction

Bandgap engineering is essential for optimizing the photocatalytic efficiency of semiconductor materials. Bismuth ferrite (BiFeO_3_, BFO), a room-temperature multiferroic perovskite, combines robust ferroelectricity (*T*_C_~1103 K) and G-type antiferromagnetism (*T*_N_~643 K), making it a compelling candidate for applications ranging from spintronics to photocatalysis [1,2]. Its narrow bandgap (~2.2–2.7 eV) and ability to absorb visible light further position it as a promising photocatalyst. However, its practical application is hindered by intrinsic limitations such as high leakage currents from oxygen vacancies, suppressed macroscopic magnetization due to spiral spin structures, and rapid electron–hole recombination which hinder its practical utility [3,4]. These defects degrade its dielectric stability, suppress magnetization, and impair charge carrier dynamics, necessitating strategies to enhance BFO’s functional properties.

Transition metal doping has emerged as an effective approach to overcoming these limitations. Cobalt (Co), in particular, is a compelling substitute due to its compatible ionic radius (Co^2+^: 0.745 Å vs. Fe^3+^: 0.645 Å) and multivalent nature, which facilitate lattice substitution at Fe^3+^ sites. It stands out for its unique ability to simultaneously tune electronic, magnetic, and structural properties [5,6]. Introducing Co^2+^/Co^3+^ ions into the BFO lattice induces critical modifications. The larger ionic radius of Co^2+^ generates lattice strain, reducing crystallite size and disrupting the antiferromagnetic cycloidal spin order to enhance weak ferromagnetism [6]. Additionally, Co doping hybridizes its three *d* orbitals with O 2*p* states, creating intermediate energy levels that narrow the bandgap and significantly improve its ability to absorb visible light [6,7]. Furthermore, aliovalent Co^2+^ doping promotes charge compensation via oxygen vacancy formation, which optimizes electrical conductivity while stabilizing the perovskite structure [8]. However, isolated doping often fails to effectively suppress photogenerated charge recombination, limiting catalytic performance.

To further enhance charge separation, heterostructure engineering, particularly at the BiFeO_3_/Bi_25_FeO_40_ interfaces, offers a promising solution by facilitating charge separation and improving electron mobility. These heterojunctions leverage interfacial charge transfer between phases to suppress electron–hole recombination, extend carrier lifetimes, and enhance redox activity [9,10]. The built-in electric field at the BiFeO_3_/Bi_25_FeO_40_ interface facilitates the efficient separation of photogenerated charges, while the synergistic alignment of band structures broadens light absorption and improves photocatalytic efficiency [11]. Additionally, the heterostructure’s increased surface-to-volume ratio exposes more active sites for catalytic reactions, further amplifying performance [9]. Conventional methods like solid-state synthesis lack the precision to achieve nanoscale homogeneity, underscoring the need for advanced fabrication techniques.

In achieving precise stoichiometric control and minimizing secondary phases, the sol–gel method is uniquely advantageous [9]. Unlike solid-state synthesis, sol–gel processing enables atomic-level homogeneity and low-temperature crystallization (~550 °C), mitigating bismuth volatilization and ensuring uniform Co incorporation [5,10]. This precision is crucial for constructing heterostructured systems, such as BiFeO_3_/Bi_25_FeO_40_ [11]. While Co substitution and heterostructure engineering have been studied individually, their combined effects on band structure modulation, defect passivation, and multiferroic enhancement remain unexplored. Specifically, the interplay between Co-induced lattice distortions, heterophase stability, and interfacial charge transfer in BiFeO_3_/Bi_25_FeO_40_ systems is lacking further investigation.

In this work, we synthesize Co-doped BiFeO_3_/Bi_25_FeO_40_ heterostructured nanopowders (x = 0.07, 0.15) via sol–gel processing, alongside pristine BFO for comparison. We systematically tune the phase composition, crystallite size, and optical properties to achieve a bandgap reduction that surpasses those of individual BFO and sillenite. Through systematic structural, vibrational, optical and dielectric analyses, we demonstrate how Co doping and heterojunction formation synergistically enhance electrical conductivity and promote band gap tuning. This study provides new insights into the correlation between Co doping, Co-doped BiFeO_3_/Bi_25_FeO_40_ heterostructure formation and band gap reduction, which might provide a framework for designing advanced multiferroic nanocomposites that are promising for environmental applications.

## 2. Materials and Methods

### 2.1. Synthesis Procedure

BiFeO_3_ and Co-doped BiFeO_3_/Bi_25_FeO_40_ heterostructured nanopowders with cobalt substitution levels of x = 0.07 and 0.15, labelled as BFO, BFCO_0.07_, and BFCO_0.15_, were synthesized using a citrate-modified sol–gel approach [11]. Stoichiometric quantities of bismuth (III) nitrate pentahydrate (Bi (NO_3_)_3_·5H_2_O), iron (III) nitrate nonahydrate (Fe (NO_3_)_3_·9H_2_O), and cobalt (II) nitrate hexahydrate (Co (NO_3_)_2_·6H_2_O) were dissolved separately in deionized water under continuous magnetic stirring. Nitric acid (HNO_3_) was added dropwise to each solution to ensure complete dissolution. The individual solutions were combined and homogenized at 25 °C for 30 min. Citric acid (C_6_H_8_O_7_) and ethylene glycol (C_2_H_6_O_2_) were introduced into the mixture in stoichiometric proportions as complexing and cross-linking agents, respectively. The solution was heated at 70 °C to induce gelation. The resulting gel was dehydrated at 100 °C for 4 h to form a xerogel, which was then ground into a fine powder and calcined at 600 °C in air for 1 h to yield crystallized nanoparticles.

### 2.2. Characterization Techniques

Crystalline phase identification and structural analysis were performed using X-ray diffraction (XRD) with a Bruker D8 Advance XR01 diffractometer (Bruker AXS GmbH, Karlsruhe, Germany) equipped with Cu Kα radiation (λ = 1.5406 Å), which includes both Kα_1_ (λ = 1.54056 Å) and Kα_2_ (λ = 1.54439 Å) components. Data were collected over a 2θ range of 20–80° with an angular resolution of 0.02°. Phase identification and lattice parameters were quantified through Rietveld refinement using the FullProf suite (version: April 2023). Moreover, to analyze bonding configurations and detect organic residuals, Fourier-transform infrared spectroscopy (FTIR) was employed. FTIR measurements were carried out using a Perkin Elmer FTIR-100 spectrometer (PerkinElmer, Waltham, MA, USA) over the spectral range of 400–4000 cm^−1^. Powder morphology was analyzed via scanning electron microscopy (SEM) using a TESCAN VEGA3 SBH microscope (TESCAN, Brno, Czech Republic) equipped with a Bruker XFlash 410M energy-dispersive spectroscopy (EDS) (Bruker Corporation, Billerica, MA, USA) detector for elemental analysis. Optical properties and bandgap energies were determined using UV-Vis spectroscopy with an Evolution 220 spectrometer (Thermo Scientific Co., Ltd., Waltham, MA, USA). For dielectric measurements, cylindrical disc-shaped pellets were fabricated, and a uniform layer of conductive silver paste was applied to both surfaces to ensure optimal electrical contact. Dielectric properties were characterized using a Wayne Kerr 6425 component analyzer (Wayne Kerr Electronics Ltd., Bognor Regis, West Sussex, UK).

## 3. Results and Discussion

### 3.1. XRD Analysis

**Figure 1:**
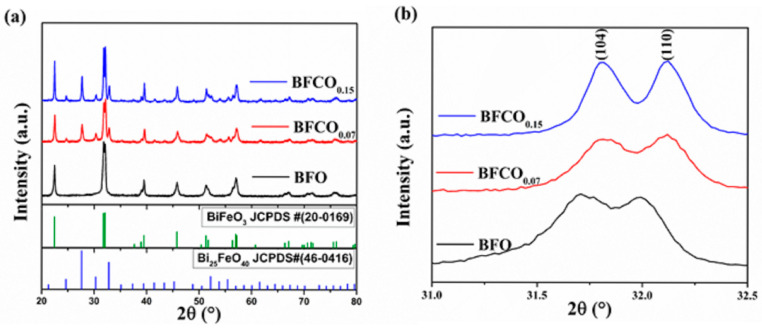
(**a**) X-ray diffraction patterns of BFO, BFCO_0.07,_ and BFCO_0.15_ along with the standard JCPDS data of BiFeO_3_ and Bi_25_FeO_40_. (**b**) Partial enlargement of the XRD patterns at 31–32.5°.

The crystal structures of the BiFeO_3_ and Co-doped BiFeO_3_/Bi_25_FeO_40_ heterostructured nanopowders (BFCO_0.07_, BFCO_0.15_) were systematically investigated using X-ray diffraction coupled with Rietveld refinement. Figure 1a displays the XRD patterns of all samples, where the sharp, high-intensity peaks with narrow full-width-at-half-maximum (FWHM) values confirm the high crystallinity of the synthesized materials. For BFO, the diffraction peaks align with the rhombohedral perovskite structure (space group R3c, JCPDS No. 20-0169) [12]. In contrast, the BFCO_0.07_ and BFCO_0.15_ samples exhibit additional peaks primarily indexed to the cubic iron–sillenite-phase Bi_25_FeO_40_ (space group I23, JCPDS No. 46-0416) [13], confirming the coexistence of perovskite (R3c) and sillenite (I23) phases. This phase segregation, modulated by cobalt doping, arises from the controlled Bi^3+^/Fe^3+^ molar ratio during synthesis, as previously reported by Wang et al. [14]. Specifically, excess Bi^3+^ promotes the formation of Bi_25_FeO_40_ alongside BiFeO_3_, while excess Fe^3+^ favours Bi_2_Fe_4_O_9_, highlighting the critical role of stoichiometric control in phase engineering.

A magnified view of the (104) and (110) Bragg reflections in Figure 1b reveal distinct peak shifts that broaden with the cobalt substitution. For BFCO_0.07_, a systematic shift toward higher angles indicates lattice contraction due to the doping of Fe^3+^ (ionic radius: 0.645 Å) by smaller Co^3+^ ions (0.545 Å). Conversely, BFCO_0.15_ shows negligible peak shifting, suggesting competing effects from larger Co^2+^ (0.745 Å) and/or oxygen vacancy formation at higher doping levels. The partial oxidation of Co^2+^ to Co^3+^ during synthesis is facilitated by nitric acid and ethylene glycol [6]. This oxidation process introduces a dual effect: initial lattice contraction (Co^3+^ doping) followed by potential expansion due to residual Co^2+^ or oxygen vacancies at higher doping levels. These observations underscore the doping-dependent interplay between ionic radius mismatch and defect chemistry.

**Figure 2:**
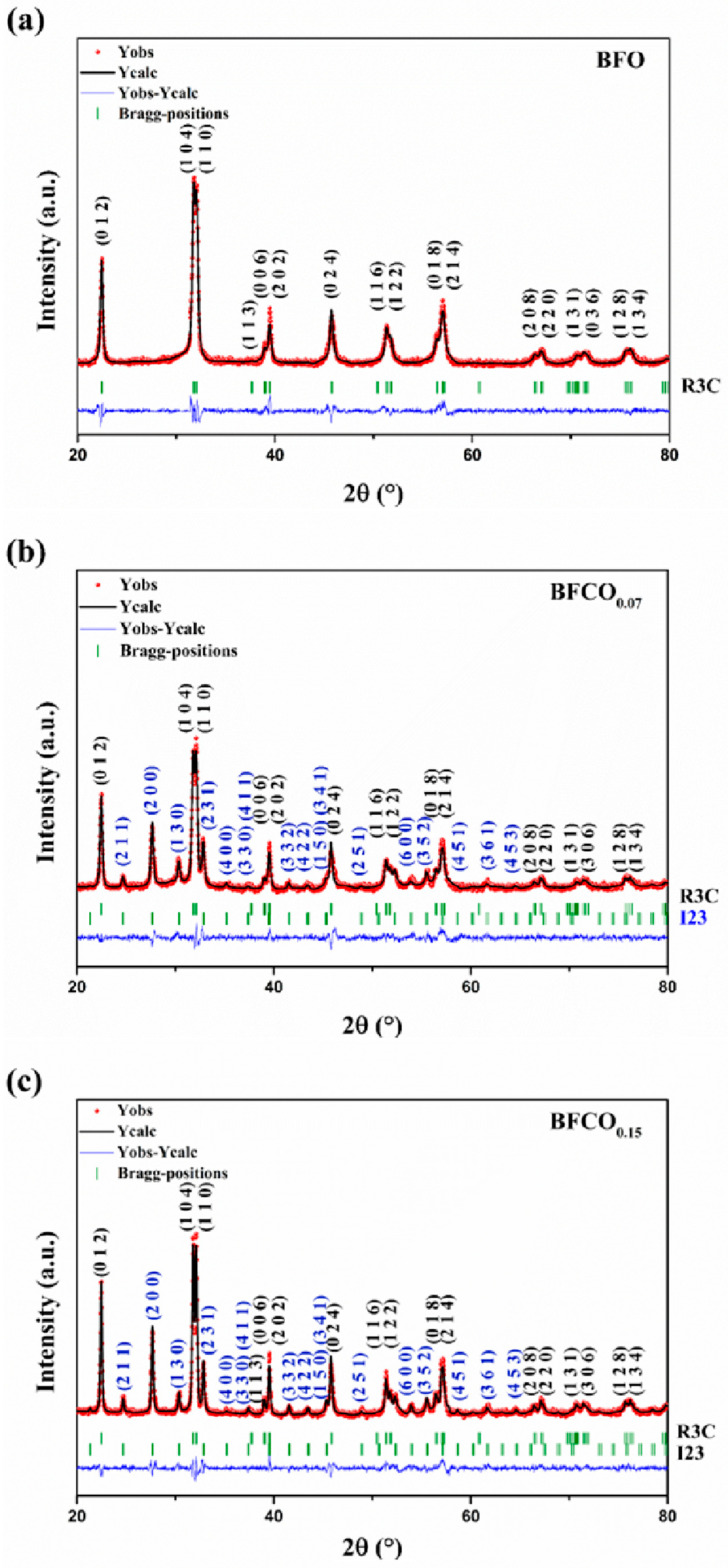
Rietveld refinement of the X-ray diffraction patterns of (**a**) BFO, (**b**) BFCO_0.07_, and (**c**) BFCO_0.15_ nanopowders.

Rietveld refinement quantitatively validated the phase composition and structural parameters of all samples, as shown in Figure 2. BFO crystallizes purely in the rhombohedral R3c phase, whereas BFCO_0.07_ and BFCO_0.15_ exhibit biphasic structures (R3c + I23), with phase ratios of ~73:27 and ~76:24, respectively. The lattice parameters and unit cell volumes decrease progressively with cobalt doping (Table 1). Although the sillenite phase was primarily identified in the study as Bi_25_FeO_40_, the progressive lattice contraction (a = 10.203 Å for BFCO_0.07_ and 10.194 Å for BFCO_0.15_) suggests that the sillenite phase corresponds to Co-doped Bi_25_FeO_40_ i.e., (Bi_25_(Fe,Co)O_40_) [15]. This aligns with partial doping of smaller Co^3+^ ions into Fe^3+^ sites, rather than the formation of distinct Bi_25_CoO_40_, which would require a stoichiometric Co:Fe ratio (1:0) and exhibit a smaller lattice parameter of (~10.13 Å) [16].

**Figure 3:**
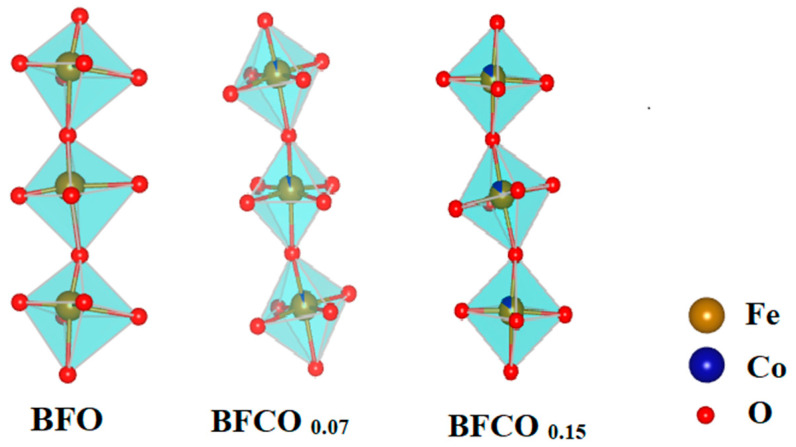
A schematic representation of bond lengths and angles in BFO, BFCO_0.07_, and BFCO_0.15_ nanopowders using Vesta software (version: 4.3.0).

Further structural insights were derived from bond lengths and angles (Table 2). In BFCO_0.07_, the shortened Fe–O bond (1.994 Å vs. 2.013 Å in BFO) and near-linear Fe–O–Fe angle (166.09°) reflect reduced octahedral tilting and compressive strain within the dominant R3c phase. Coherent interfaces with the cubic I23 phase (27% fraction) further alleviate interfacial strain. In contrast, BFCO_0.15_ exhibits elongated Fe–O bonds (2.009 Å) and a distorted Fe–O–Fe angle (160.23°), indicative of tensile strain and enhanced octahedral tilting. While the overall lattice parameters indicate compressive strain in the perovskite phase due to lattice mismatch at the R3c/I23 heterointerface, local atomic distortions within the perovskite phase, particularly in BFCO_0.15_, show evidence of tensile strain. This local tensile strain, evidenced by elongated Fe–O bonds (2.009 Å) and distorted Fe–O–Fe angles (160.23°), arises from the incorporation of larger Co^2+^ ions (0.745 Å) and potential oxygen vacancies, which cause local lattice expansion despite the overall compressive trend [17]. A schematic presentation of bond lengths and angles in all samples using Vesta software is depicted in Figure 3.

To deconvolute the contributions of crystallite size and lattice strain to X-ray diffraction peak broadening, a Williamson–Hall (W-H) analysis was employed. The W-H method assumes that these two factors independently contribute to the full-width-at-half-maximum (FWHM, β) of Bragg reflections, with their combined effects expressed as:

(1) $$\beta =\beta_{strain}+\beta_{size}$$

By incorporating Bragg’s law (nλ = 2d sinθ) and approximating the strain as a function of sinθ, the relationship is linearized into [18]:

(2) $$\beta cos\theta =4\varepsilon *sin\theta +\frac{k\lambda }{D}$$

Here, β (in radians) is the measured FWHM, θ is the Bragg angle, k is a dimensionless shape factor (~0.9 for spherical crystallites), λ is the X-ray wavelength (1.5406 Å for Cu-Kα), D is the volume-weighted crystallite size, and ε represents the lattice microstrain.

**Figure 4:**
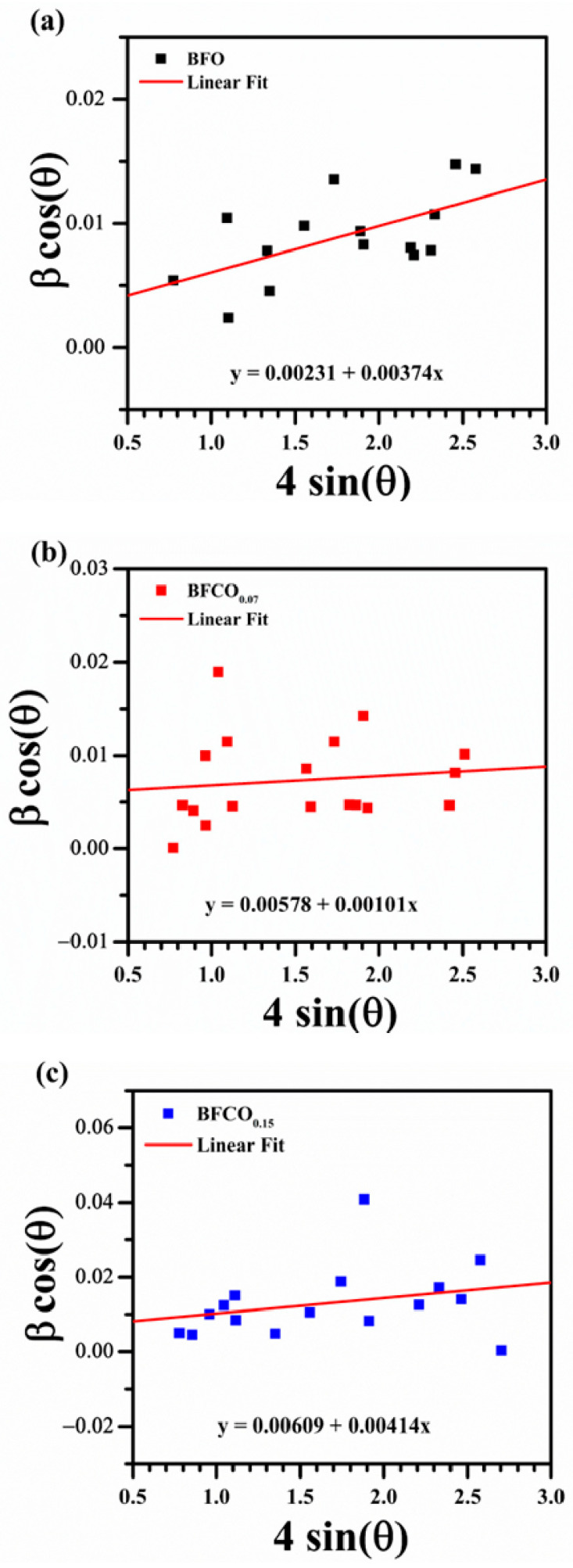
Williamson–Hall plots for (**a**) BFO, (**b**) BFCO_0.07_, and (**c**) BFCO_0.15_.

To quantify D and ε, a W-H plot was constructed by graphing β cosθ (*y*-axis) against 4sinθ (*x*-axis). The slope of the linear fit corresponds to ε, while the y-intercept yields D via D = kλ/(intercept). To ensure the accuracy of the Williamson–Hall (W-H) analysis, the pseudo-Voigt function was employed to extract FWHM values. While the W-H plot (Figure 4) might appear scattered, which is inherent to polycrystalline systems with strain anisotropy, the linear regression yielded a goodness-of-fit value which confirms dominant isotropic contributions. The extracted D and ε values for BFO, BFCO_0.07_, and BFCO_0.15_ are compiled in Table 3.

The Williamson–Hall (W-H) analysis quantified the crystallite size and microstrain evolution. BFO exhibits moderate tensile strain (3.74 × 10^−3^), intrinsic to its rhombohedral structure [19]. BFCO_0.07_ shows a significantly reduced strain (1.01 × 10^−3^), attributed to Co^3+^ induced lattice contraction and probably interfacial strain buffering by the I23 phase [20]. However, BFCO_0.15_ exhibits heightened strain (4.14 × 10^−3^), surpassing even undoped BFO, due to Co^2+^-mediated lattice expansion, oxygen vacancies, and possibly to the diminished I23 phase contribution [20,21]. The excellent fit of the Rietveld refinement for BFCO_0.07_ and BFCO_0.15_ in addition to the reduced microstrain in BFCO_0.07_ suggest well-integrated BiFeO_3_ and sillenite phases, potentially forming coherent interfaces. For BFCO_0.15_, the higher microstrain and lower sillenite content (23.68%) may indicate less coherence, but the heterostructure persists. A heterointerface is presumed to form between low-index planes based on similar d-spacings consistent with general principles in ceramic heterostructures [11,22].

The crystal structure of Co-doped BiFeO_3_/Bi_25_FeO_40_ heterostructured nanopowders is influenced by strain from two sources: (1) interfacial lattice mismatch between the perovskite (R3c) and sillenite (I23) phases, and (2) Co doping. The mismatch between the R3c (a ≈ 5.58 Å) and I23 (a ≈ 10.2 Å) phases induces compressive strain in the perovskite and tensile strain in the sillenite at their interface, as evidenced by lattice contraction (Table 1: R3c a-axis from 5.583 Å in BFO to 5.572 Å in BFCO_0.15_; I23 a-axis from 10.203 Å in BFCO_0.07_ to 10.194 Å in BFCO_0.15_). In BFCO_0.07_, Co^3+^ substitution (ionic radius 0.545 Å) enhances this compression, reducing microstrain (1.01 × 10^−3^ vs. 3.74 × 10^−3^ in BFO) and shortening Fe–O bonds (1.994 Å vs. 2.013 Å in BFO), with the I23 phase (27%) buffering interfacial stress. In contrast, BFCO_0.15_ shows higher microstrain (4.14 × 10^−3^) due to lower I23 content (24%) and local tensile strain within the perovskite phase, caused by larger Co^2+^ ions (0.745 Å) and oxygen vacancies, leading to elongated Fe–O bonds (2.009 Å) and distorted Fe–O–Fe angles (160.23°). These strain effects, supported by similar BiFeO_3_-based heterostructures [17,23], enhance bandgap tuning and electrical properties.

These findings underscore the delicate balance required to optimize multifunctional performance in perovskite–sillenite heterostructured nanopowders.

### 3.2. FTIR Analysis

**Figure 5:**
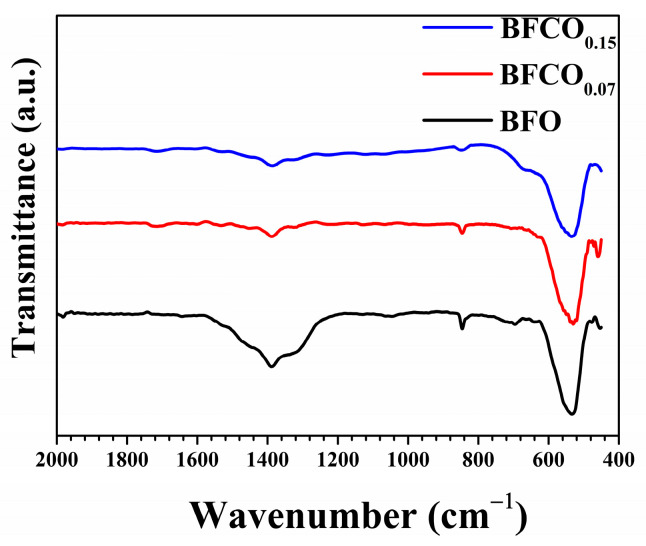
FTIR spectra of BFO, BFCO_0.07_, and BFCO_0.15_ nanopowders.

The Fourier-transform infrared (FTIR) spectra of pristine BFO and Co-doped BiFeO_3_/Bi_25_FeO_40_ heterostructured nanopowders, shown in Figure 5, reveal critical insights into their structural and compositional characteristics. Initial observations of the FTIR spectra reveal distinctive features present for all samples, which are a broad absorption band spanning 450–600 cm^−1^ that confirms the perovskite framework, with distinct vibrational modes attributed to metal–oxygen bonding [24]. For undoped BFO, the prominent peak at 474 cm^−1^ corresponds to Fe-O stretching vibrations in octahedralFeO_6_, while a secondary peak at 538 cm^−1^ aligns with symmetric O-Fe-O bending modes [25,26]. Notably, the presence of a distinct peak at 810 cm^−1^ in BFO materials reflects their structural crystallinity [27]. The detection of a consistent peak near 845 cm^−1^ in all our samples confirms their high crystallinity, which aligns closely with the XRD results. Moreover, the Bi-O stretching vibrations are typically observed near 1000 cm^−1^ in BFO samples [27,28]. In our work, Bi-O stretching vibrations are detected at about 1045 cm^−1^ in the BFO sample, while they were obscured in Co-doped BiFeO_3_/Bi_25_FeO_40_ samples due to Co^3+^ doping-induced lattice distortions. Residual nitrate ions (NO_3_^−^), identified between 1325 and 1390 cm^−1^ [29], and traces of ethylene glycol (C-H bending at 1476 cm^−1^), as reported in analogous studies [25,26]. The weak absorption band from 1500 cm^−1^ to 1646 cm^−1^ is indicative of the presence of water molecules [26,30]. In BFCO_0.07_, Co^3+^ doping (ionic radius: 0.545 Å) induces lattice contraction (Fe–O: 1.994 Å) and compressive strain (1.01 × 10^−3^), manifesting as a slight redshift in the O–Fe–O bending mode (from 538 to 535 cm^−1^) and peak broadening due to interfacial strain buffering in the cubic sillenite phase (I23, 27% phase fraction) [24,25]. Conversely, BFCO_0.15_ exhibits a relatively pronounced redshift compared to BFO (from 538 to 530 cm^−1^) and a diminished Fe–O intensity, aligning with XRD-derived bond elongation (Fe–O: 2.009 Å), tensile strain (4.14 × 10^−3^), and octahedral distortion (Fe–O–Fe angle: 160.23°) caused by partial Co^2+^ (0.745 Å) incorporation and oxygen vacancies [5]. Interfacial strain is evidenced by correlated R3c/I23 lattice contractions (Table 1) and IR spectral shifts. The redshift in O–Fe–O bending modes from 538 to 530 cm^−1^ reflects compressive strain in the perovskite phase due to lattice mismatch with the cubic sillenite phase. This strain is buffered by the sillenite phase (24% fraction), which reduces interfacial tension, as observed in analogous composites [31]. Such strain transfer might reflect the dielectric/optical trends of the material which is consistent with prior studies on strained BiFeO_3_ heterostructures [32]. The cubic sillenite phase, while prominent in XRD (~24–27%), lacks distinct IR signatures (e.g., 578 cm^−1^ Fe–O stretch) due to spectral overlap with perovskite modes or weak IR activity, as observed in analogous Bi_25_FeO_40_/Bi_2_Fe_4_O_9_ composites [33].

### 3.3. Microstructural Analysis

**Figure 6:**
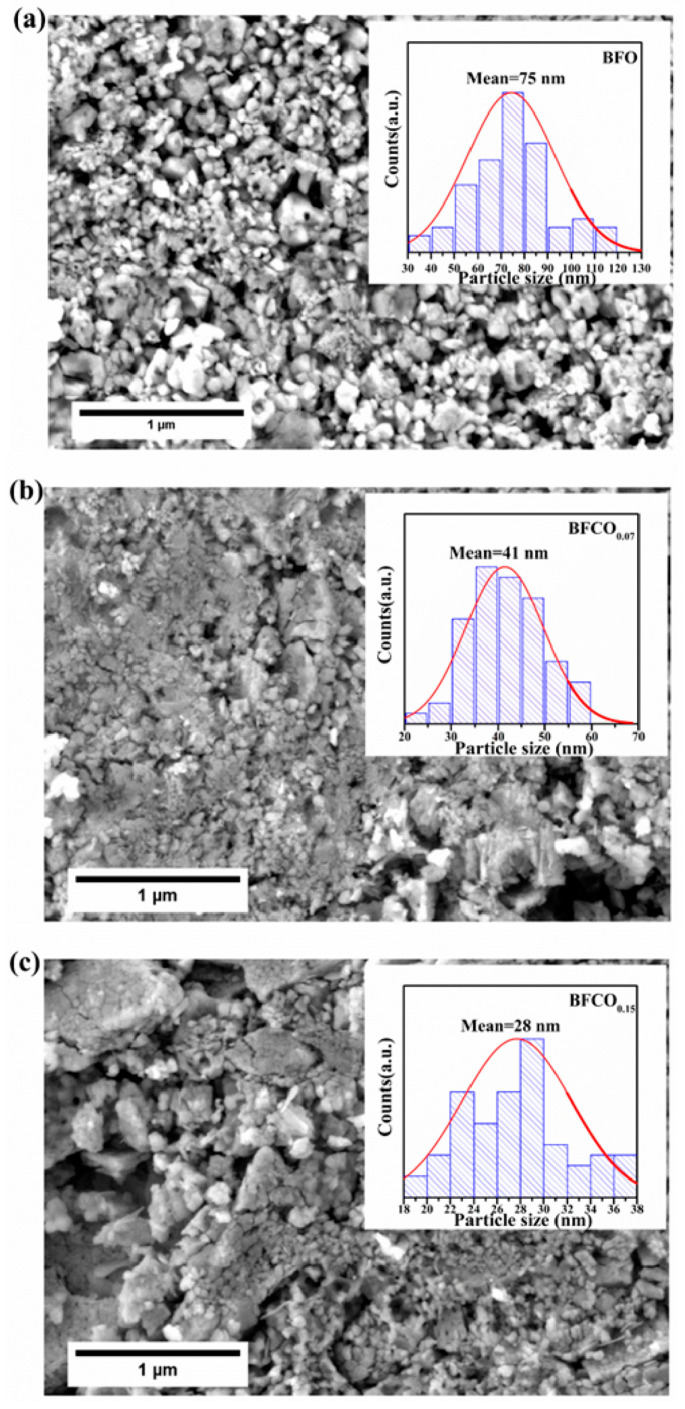
FESEM micrographs of (**a**) BFO, (**b**) BFCO_0.07_, and (**c**) BFCO_0.15_ nanopowders with the corresponding average grain size distribution shown in the inset.

Field emission scanning electron microscopy (FE-SEM) reveals significant morphological and microstructural evolution in BFO, BFCO_0.07_, and BFCO_0.15_ nanopowders as a function of cobalt doping, as shown in Figure 6a. BFO consists of homogeneous, spherical particles averaging 75 nm in diameter, as shown in Figure 6a. With cobalt doping, BFCO_0.07_ and BFCO_0.15_ exhibit a monotonic reduction in particle size to ~28 nm, as shown in Figure 6b,c, along with increased agglomeration and a narrower size distribution as depicted in the insets in the figures. This size reduction aligns with the XRD-derived crystallite dimensions estimated from the Williamson–Hall analysis (~23–24 nm). The biphasic heterostructures (phase ratios R3c-I23 of 73:27 and 76:24) of BFCO_0.07_ and BFCO_0.15_, respectively, promote interphase agglomeration, as coherent R3c-I23 interfaces was found to reduce interfacial strain.

The size reduction follows doping-dependent mechanisms: In BFCO_0.07_, the substitution of Fe^3+^ (0.645 Å) by smaller Co^3+^ (0.545 Å) induces lattice contraction, while the 27% I23 phase buffers interfacial strain, collectively limiting crystallite growth [6]. In BFCO_0.15_, a reduced I23 phase content (24%) leads to diminished strain accommodation, allowing tensile strain from partial Co^2+^ substitution (0.745 Å) and oxygen vacancies to dominate. This strain distorts the lattice, despite the smaller crystallites. Agglomeration intensifies in doped samples due to the increased nanoscale surface energy [34].

**Figure 7:**
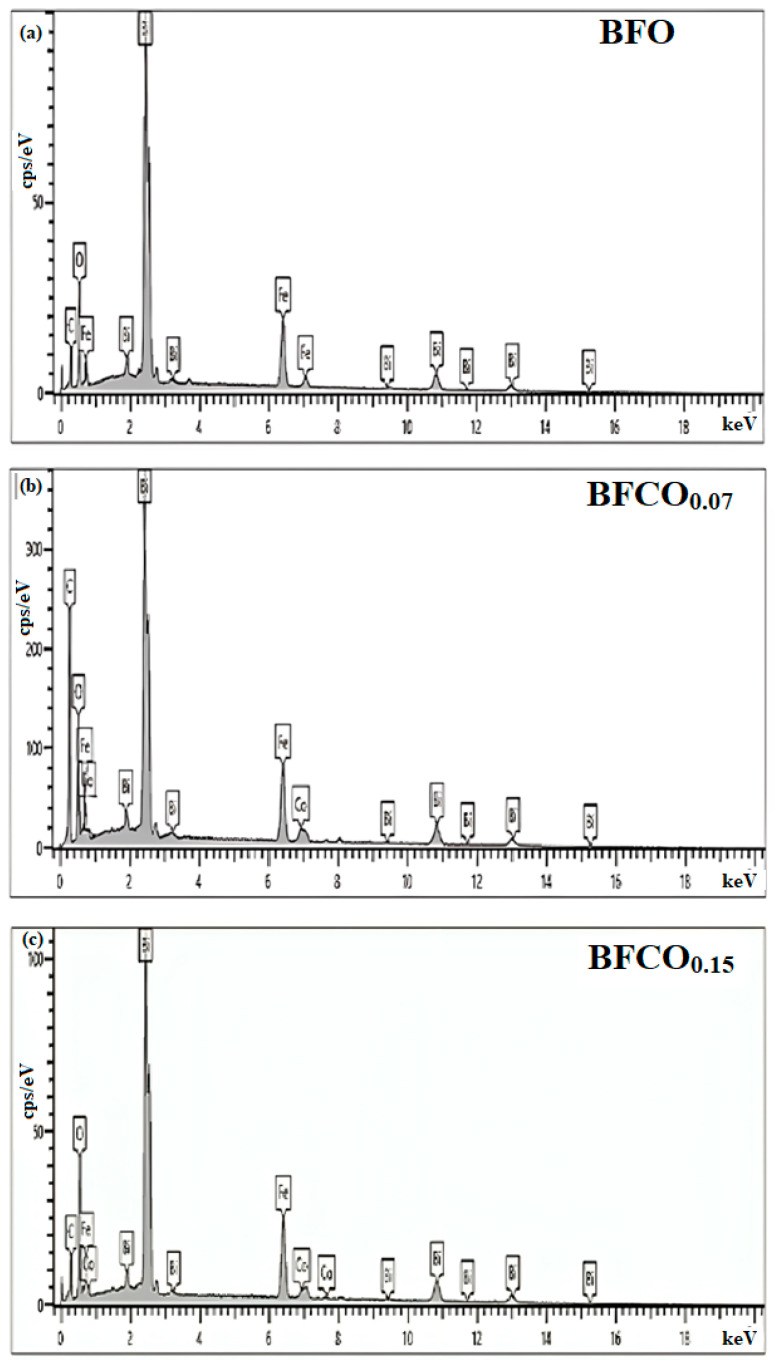
EDX spectra of BFO, BFCO_0.07_, and BFCO_0.15_ nanopowders.

Energy-dispersive X-ray spectroscopy (EDX), depicted in Figure 7, confirmed the elemental composition and phase purity of the nanoparticles. The spectra show Bi, Fe, Co, and O across all samples, with characteristic peaks at O (K_α_, 0.52 keV), Fe (L_α_, K_α_, K_β_ at 0.70, 6.40, and 7.05 keV), Bi (M_α_, L_α_, L_β_ at 2.49, 10.38, and 13.02 keV), and Co (K_α_, L_α_ at 6.93 and 0.77 keV). No extraneous peaks were detected, confirming phase purity.

The reduced particle size and increased surface area in BiFe_1−x_Co_x_O_3_/Bi_25_(Fe,Co)O_40_ heterostructured nanopowders may enhance light absorption and active sites for redox reactions. Coupled with the R3c-I23 heterojunction, which facilitates charge separation at phase interfaces, this could improve photocatalytic efficiency, as reported for similar biphasic oxides [14].

### 3.4. UV–Visible Analysis

**Figure 8:**
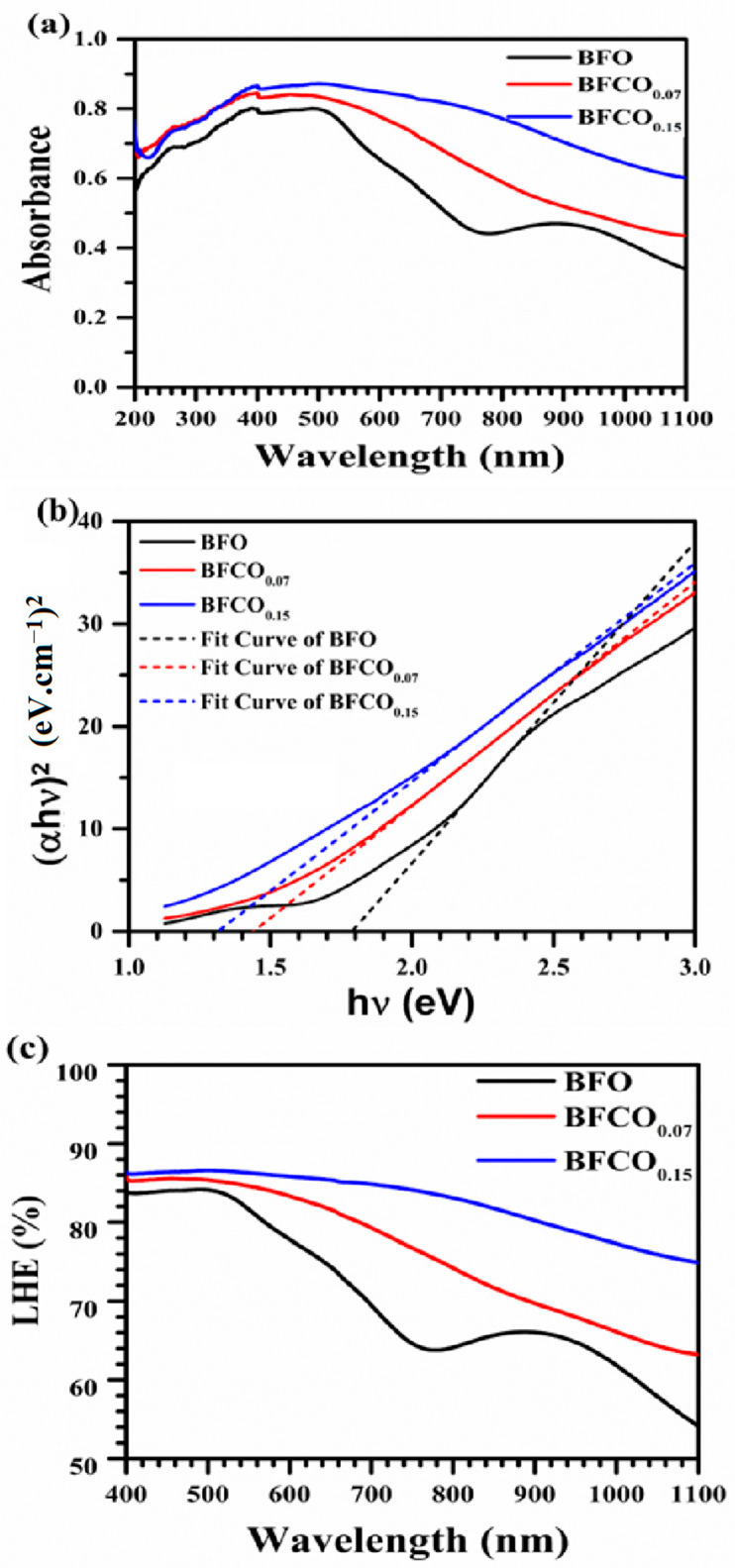
(**a**) Absorbance spectra of composition. (**b**) Tauc’s plots to extract the optical bandgap. (**c**) Light-harvesting efficiency of BFO, BFCO_0.07_, and BFCO_0.15_ nanopowders.

The optical absorption properties of BFO, BFCO_0.07_, and BFCO_0.15_ heterostructured nanopowders were examined to investigate the synergistic impact of phase segregation and cobalt doping on bandgap modulation. Figure 8a presents the UV–Vis absorption spectra of BFO, BFCO_0.07_, and BFCO_0.15_. An initial examination of Figure 8a clearly indicates that BFCO_0.07_ and BFCO_0.15_ exhibit modified optoelectronic responses relative to BFO. These changes correlate with the biphasic perovskite–sillenite structure, lattice strain, and morphology tuned by substitution. For clarity, UV-Vis measurements were performed in direct transmission mode on consolidated pellets (1 mm thickness and 8 mm diameter) to ensure reproducibility. Absorption coefficients (α) were calculated using the Beer–Lambert law (α = 2.303 A/t, where t = 1 mm) [5,35]. BFO nanoparticles show moderate absorption in the 200–770 nm range with a peak of approximately 80%. This behaviour reflects its rhombohedral perovskite structure and inherent bandgap. In contrast, cobalt-doped samples display a redshift in the absorption edge and enhanced absorption in the visible range. For BFCO_0.15_, the absorption exceeds 90% at 400–770 nm. This enhancement arises from Co doping and interfacial strain between the perovskite (R3c) and sillenite (I23) phases. Co^3+^ substitution (ionic radius 0.545 Å) and partial Co^2+^ incorporation in BFCO_0.15_ introduce new electronic states, while lattice mismatch (R3c: a ≈ 5.58 Å; I23: a ≈ 10.2 Å) induces compressive strain in the perovskite phase, narrowing the bandgap from 1.78 eV in BFO to 1.31 eV in BFCO_0.15_ [17,23]. Critically, XRD refinement (Figure 2) confirms the absence of Bi_25_CoO_40_, ensuring the optical response originates from the R3c perovskite and sillenite phases.

The optical bandgap (E_g_) was derived from the Tauc relation for direct transitions:

(3) $$(\alpha h\vartheta )=A{\left(h\vartheta -E_{g}\right)}^{n}$$

where hϑ is the photon energy [36].

Bandgap values were determined exclusively via Tauc plots [(αhν)^2^ vs. hν], as consolidated pellets were analyzed in absorption mode. This method directly correlates the absorption edge to the dominant R3c phase, even in biphasic systems [22].

BiFeO_3_ shows a direct bandgap of 1.78 eV due to Fe^3+^-O 3d–p charge transfer in its R3c phase [5,37]. Cobalt incorporation on the other side shifts the absorption edge toward a lower energy. The bandgap decreases to 1.42 eV for BFCO_0.07_ and to 1.31 eV for BFCO_0.15_, as confirmed by the Tauc plots in Figure 8b. This reduction reflects Co^3+^ 3d^6^ mid-gap states, tensile strain (evidenced by XRD lattice contraction), and heterojunction-driven charge separation between the R3c and I23 phases.

The reduction in bandgap could be attributed to correlated factors, namely, Co^3+^ substitution at the Fe^3+^ site might introduce intermediate 3d^6^ states below the conduction band [38]. In addition, tensile lattice strain and oxygen vacancies at higher doping levels might further perturb the Fe/Co–O hybridization [39]. Furthermore, particle size reduction (from 75 nm in BiFeO_3_ to 28 nm in BFCO_0.15_) could increase the surface-to-volume ratio, which might enhance the defect-mediated absorption [40].

Taking the above into account, the low band gap energy of BFCO_0.15_ could be attributed to disorder-induced bandgap narrowing due to the tensile strain [41]. In addition, oxygen vacancies and Co defect states might introduce mid-gap levels that counteract strain-induced shifts [39,42]. Notably, the heterointerface between the R3c and sillenite phases enhances light trapping and charge transfer, as evidenced by dielectric enhancement (Figure 6a) and prior studies on similar systems [14].

In the same context, Figure 8c reveals that the light-harvesting efficiency can be estimated as [43]:

(4) $$\mathrm{LHE}=1-10^{-A}$$

where A is the material’s absorbance, which was found to improve accordingly. As show in Figure 8c, BFCO_0.15_ attains an LHE greater than 90% for wavelengths above 600 nm, compared with 72% for BiFeO_3_ nanoparticles. This improvement is probably linked to enhanced visible-to-NIR photon capture through interfacial exciton dissociation [44]. The observed 26% reduction in bandgap and the high LHE in BFCO_0.15_ is indicative of its promising photocatalytic performance [5,38].

Bandgap engineering in these systems results from phase segregation, defect chemistry, and morphological evolution. In BFCO_0.07_, compressive strain and shortened Fe–O bonds promote delocalized charge carriers, while the I23 phase (27% fraction) reduces interfacial recombination [45]. In BFCO_0.15_, tensile strain and elongated Fe–O bonds enhance defect-assisted absorption despite increased charge trapping [46].

This work establishes Co-doped BiFeO_3_/Bi_25_FeO_40_ heterostructures as tunable optoelectronic platforms. Strain-engineered band alignment, biphasic synergy, and nanoscale agglomeration improve light absorption and charge separation, which are crucial for photocatalysis and photovoltaic applications [47].

### 3.5. Dielectric Analysis

**Figure 9:**
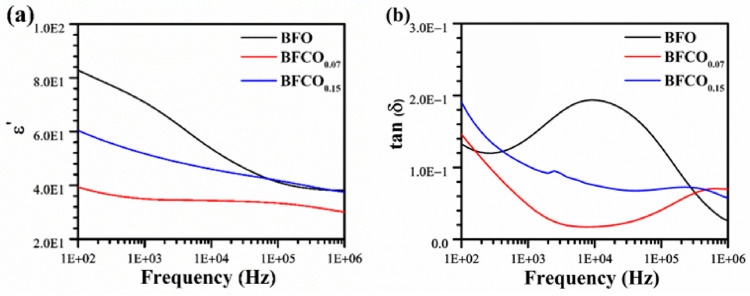
Variation in dielectric properties of BFO, BFCO_0.07_, and BFCO_0.15_ as a function of frequency at room temperature: (**a**) real dielectric constant and (**b**) the loss factor tan δ.

The dielectric properties of Co-doped BiFeO_3_/Bi_25_FeO_40_ heterostructures exhibit frequency-dependent behaviour governed by interfacial polarization, defect dynamics, and structural evolution. Figure 9a illustrates the variation in permittivity as a function of frequency at room temperature for pure BFO and cobalt-doped samples BFCO_0.07_ and BFCO_0.15_.

It can be seen that, for all samples, the overall dielectric permittivity decreases with increasing frequency, a typical behaviour of ferroelectric materials consistent with Maxwell–Wagner–Sillars (MWS) polarization [48,49].

At low frequencies, all samples experience a peak in their real permittivity (ε′) values; this observation is attributed to the collective contribution of different polarization mechanisms including space charge, dipolar, and ionic polarization. However, as the frequency increases, the ability of charge carriers to follow the rapid variations in the electric field diminishes, leading to a gradual decrease in permittivity until a plateau is reached at high frequency, where only electronic and dipolar contributions remain.

Pure BFO exhibits the highest permittivity at low frequencies, which arises from oxygen vacancy (V_O_^••^) migration. The doping of 7 mol% Co to BiFeO_3_/Bi_25_FeO_40_ leads to a significant reduction in permittivity. This reduction stems from two synergistic factors:

- [Co^2+^-V_O_^••^] defect dipole formation in the perovskite phase, which traps charges, restricts charge carrier mobility, and reduces space charge polarization [50,51].
- The intrinsic dielectric behaviour of the (I23) sillenite phase (~27% in BFCO_0.07_, Table 1), which exhibits a markedly lower permittivity (~60–100 at 1 kHz) compared to pristine BFO (~200–300) due to its cubic symmetry and reduced ionic/polarization activity [52]. The sillenite phase dilutes the overall ε′ and suppresses oxygen vacancy migration, aligning with the diminished low-frequency permittivity.

In contrast, at 15 mol% doping, a partial increase in permittivity is observed, reflecting competing effects:

- Reduced V_O_^••^ density due to Co^3+^ substitution and new defect dipoles from residual Co^2+^ [53].
- Tensile strain from the sillenite phase enhances dipolar polarization in the perovskite lattice [32], and the slight increase in perovskite content (73% (BFCO_0.07_) vs. 76.32% (BFCO_0.15_)) amplifies its contribution to ε′.

Figure 9b presents the evolution of dielectric losses (tan δ) as a function of frequency for pure BiFeO_3_ (BFO) and the cobalt-doped samples BFCO_0.07_ and BFCO_0.15_. At low frequencies (100 Hz–1 kHz), the dielectric loss peak is due to ionic/space-charge conduction. As the frequency increases, tan δ gradually decreases until it reaches the minimum value. This can be explained by the reduced contribution of interfacial polarization and space charge conduction, as charge carriers can no longer follow the rapidly alternating field. Beyond 100 kHz, tan δ slightly increases again, indicating the activation of electronic mechanisms such as dipolar relaxation and transitions between localized electronic states.

Regarding the effect of cobalt substitution, pure BFO exhibits the highest dielectric losses, especially at low frequencies, due to the high concentration of oxygen vacancies and mobile charge carriers [54]. With Co doping, dielectric losses are significantly reduced, not only due to decreased charged defects ([Co^2+^-V_O_^••^] trapping) but also because the low-loss sillenite phase (~27–28%) suppresses bulk ionic conduction.

**Figure 10:**
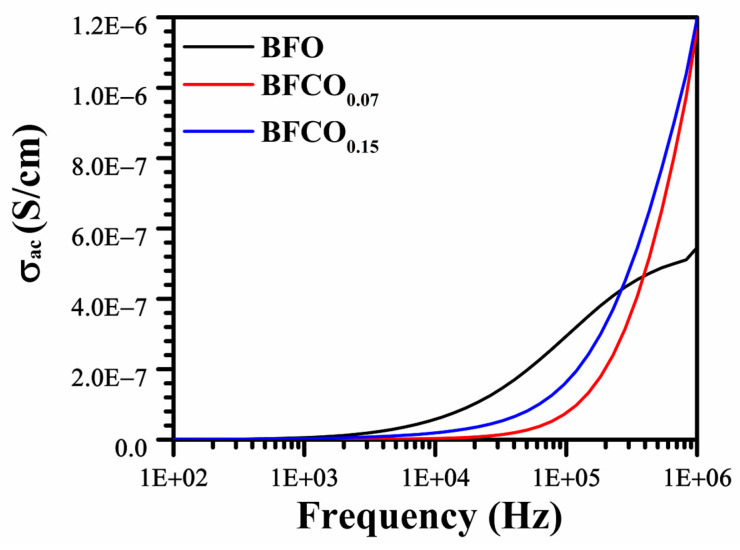
AC conductivity vs. frequency curves for pure BFO, BFCO_0.07_, and BFCO_0.15_ at room temperature.

Figure 10 illustrates the evolution of AC conductivity as a function of frequency and the cobalt doping rate, highlighting the modifications in conduction mechanisms induced by these parameters.

The AC conductivity (σ_AC_) behaviour follows Jonscher’s power law (σ_AC_∝ω*^n^*), where frequency exponent, *n*, values of 0.42 (BFO), 1.05 (BFCO_0.07_), and 0.81 (BFCO_0.15_) reveal distinct conduction mechanisms.

- Perovskite-dominated conduction: Low *n* = 0.42 in BFO indicates small-polaron hopping (Fe^2+^/Fe^3+^ pairs) [55].
- Defect dipole and sillenite effects: *n* = 1.05 in BFCO_0.07_ reflects suppressed polaron hopping (Co^3+^-mediated V_O_^••^ reduction) and the sillenite phase’s low ionic conductivity, which limits bulk conduction [56].
- Mixed mechanisms in BFCO_0.15_: Intermediate *n* = 0.81 signifies tensile strain-enhanced delocalization in the perovskite phase and residual Co^2+^-induced localized hopping [57].

These results highlight the interplay between defect chemistry, phase boundaries, and hopping mechanisms as a result of cobalt doping.

### 3.6. Impedance Analysis

**Figure 11:**
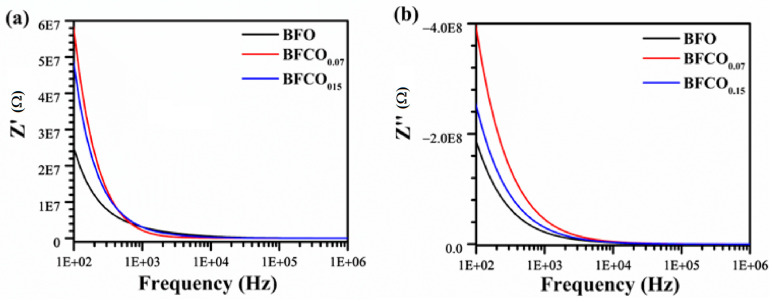
Frequency dependence of (**a**) real and (**b**) imaginary impedance of pure BFO, BFCO_0.07_, and BFCO_0.15_ at room temperature.

An analysis of Figure 11a,b illustrates the evolution of impedance as a function of frequency for the BFO, BFCO_0.07_, and BFCO_0.15_ samples. The impedance analysis reveals a progressive decrease in the real impedance (Z′) with increasing frequency for all compositions. Additionally, the curves for BFCO_0.07_ and BFCO_0.15_ exhibit a higher low-frequency Z′ for BFCO_0.07_ due to [Co^2+^-V_O_^••^] trapping, while BFCO_0.15_′s lower Z′ reflects Co^3+^-mediated V_O_^••^ reduction [58].

**Figure 12:**
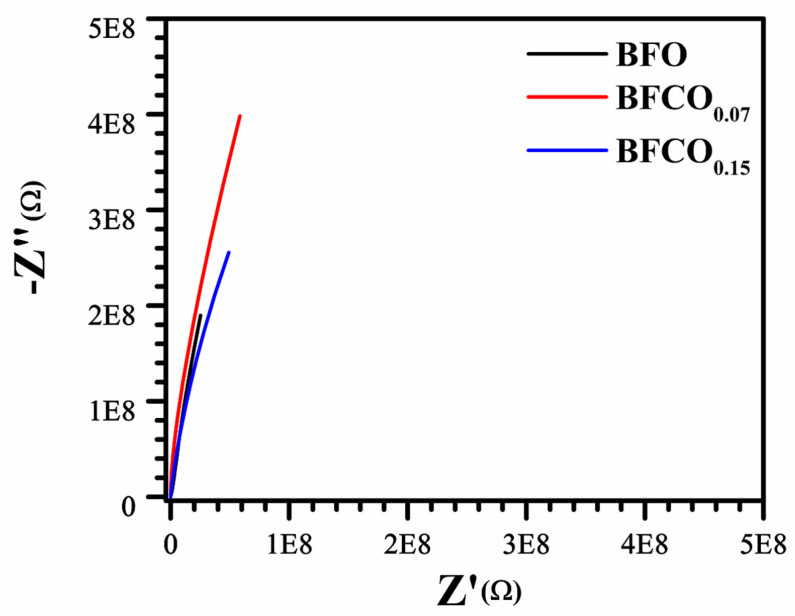
Nyquist plots of real (Z’) versus imaginary (Z’’) impedance of BFO, BFCO_0.07_, and BFCO_0.15_.

Additionally, Figure 12 presents the Nyquist plot evolution as a function of the substitution rate.

The Nyquist plots for BFO, BFCO_0.07_, and BFCO_0.15_ reveal a doping-dependent evolution in grain boundary resistance and conduction mechanisms, intricately linked to structural heterogeneity and defect chemistry. For BFCO_0.07_, the enlarged semicircle radius signifies elevated grain boundary resistance, attributed to the formation of [Co^2+^-V_O_^••^] defect dipoles that localize charges at R3c-I23 heterointerfaces [50,53]. This trapping effect is amplified by Co^3+^ doping-induced lattice contraction and agglomerated nanoparticles [51]. In contrast, BFCO_0.15_ exhibits a diminished semicircle radius despite higher Co doping, reflecting reduced grain boundary resistance due to the Co^3+^-mediated suppression of oxygen vacancies [53] and tensile strain from elongated Fe–O bonds and distorted Fe–O–Fe angles, which promote delocalized hopping [57]. The depressed, non-ideal semicircles across all samples arise from distributed relaxation times, a hallmark of structural disorder in biphasic R3c-I23 systems [49] and mixed Co^2+^/Co^3+^ valence states [50]. The convergence of the impedance curves at high frequencies depicted in Figure 11 underscores intrinsic lattice conduction, minimally perturbed by doping, while the low-frequency intercepts correlate with total resistance trends. These findings collectively demonstrate that cobalt doping in BFO heterostructures modulates interfacial polarization and defect dynamics, enabling tailored control over grain boundary-dominated conduction pathways.

## 4. Conclusions

This study demonstrates the transformative potential of Co-doped BiFeO_3_/Bi_25_FeO_40_ heterostructured nanopowders (x = 0.07, 0.15), synthesized via sol–gel processing, in advancing multiferroic nanomaterials. Co doping and biphasic engineering (R3c perovskite and I23 sillenite phases) synergistically reduce the bandgap by 26%, from 1.78 eV in pristine BiFeO_3_ to 1.31 eV at x = 0.15, while enhancing visible light absorption. Structural analyses reveal that Co^2+^/Co^3+^ doping and oxygen vacancies drive lattice strain and defect-mediated charge transport, as evidenced by frequency-dependent dielectric suppression. These tailored heterojunctions, with optimized interfacial strain, significantly boost optoelectronic performance, unlocking new possibilities for efficient photocatalysis and photovoltaics. Our findings highlight the critical interplay of doping, phase segregation, and nanoscale morphology, offering a robust framework for designing advanced multiferroic nanocomposites. Future work could explore higher doping levels or alternative transition metals to further tune nanocomposites’ functionality for energy and environmental applications.

## Figures and Tables

**Table 1:** Rietveld refinement parameters for BFO, BFCO_0.07_, and BFCO_0.15_: lattice constants, space group, and agreement factors.

Material	BFO	BFCO_0.07_		BFCO_0.15_	
Space Group	R3c	R3c	I23	R3c	I23
a (Å)	5.583	5.573	10.203	5.572	10.194
b (Å)	5.583	5.573	10.203	5.572	10.194
c (Å)	13.865	13.844	10.203	13.845	10.194
V (Å^3^)	374.382	372.484	1062.237	372.361	1059.604
Phase %	100%	73.00%	27.00%	76.32%	23.68%
R_wp_	18.2	21.3		19.9	
R_e_	13.5	16.5		14.9	
Χ^2^	1.805	1.662		1.784	

**Table 2:** Comparison of Fe–O bond lengths (Å) and Fe–O–Fe angles (°) in BFO, BFCO_0.07_, and BFCO_0.15_.

Material	BFO	BFCO_0.07_	BFCO_0.15_
Distance Fe-O (Å)	3 × Fe-O = 2.315 3 × Fe-O = 1.712 <Fe-O> = 2.013	3 × Fe-O = 2.128 3 × Fe-O = 1.860 <Fe-O> = 1.994	3 × Fe-O = 2.099 3 × Fe-O = 1.919 <Fe-O>=2.009
Angle Fe-O-Fe (°)	Fe-O-Fe = 159.83°	Fe-O-Fe = 166.09°	Fe-O-Fe = 160.23°

**Table 3:** Comparison of the lattice strain and crystallite size of BFO, BFCO_0.07_, and BFCO_0.15_.

	Strain (e) × 10^−3^	Crystallite Size (D) (nm)
BFO	3.74	60.02
BFCO_0.07_	1.01	23.99
BFCO_0.15_	4.14	22.76

## Data Availability

The original contributions presented in this study are included in the article. Further inquiries can be directed to the corresponding author.
